# Woody Species Diversity in Forest Plantations in a Mountainous Region of Beijing, China: Effects of Sampling Scale and Species Selection

**DOI:** 10.1371/journal.pone.0115038

**Published:** 2014-12-29

**Authors:** Yuxin Zhang, Shuang Zhang, Keming Ma, Bojie Fu, Madhur Anand

**Affiliations:** 1 State Key Laboratory of Urban and Regional Ecology, Research Center for Eco-Environmental Sciences, Chinese Academy of Sciences, Beijing, 100085, China; 2 Global Ecological Change Laboratory, School of Environmental Sciences, University of Guelph, Guelph, Ontario, N1G 2W1, Canada; Key Laboratory of Tropical Forest Ecology, Xishuangbanna Tropical Botanical Garden, Chinese Academy of Sciences, China

## Abstract

The role of forest plantations in biodiversity conservation has gained more attention in recent years. However, most work on evaluating the diversity of forest plantations focuses only on one spatial scale; thus, we examined the effects of sampling scale on diversity in forest plantations. We designed a hierarchical sampling strategy to collect data on woody species diversity in planted pine (*Pinus tabuliformis* Carr.), planted larch (*Larix principis-rupprechtii* Mayr.), and natural secondary deciduous broadleaf forests in a mountainous region of Beijing, China. Additive diversity partition analysis showed that, compared to natural forests, the planted pine forests had a different woody species diversity partitioning pattern at multi-scales (except the Simpson diversity in the regeneration layer), while the larch plantations did not show multi-scale diversity partitioning patterns that were obviously different from those in the natural secondary broadleaf forest. Compare to the natural secondary broadleaf forests, the effects of planted pine forests on woody species diversity are dependent on the sampling scale and layers selected for analysis. Diversity in the planted larch forest, however, was not significantly different from that in the natural forest for all diversity components at all sampling levels. Our work demonstrated that the species selected for afforestation and the sampling scales selected for data analysis alter the conclusions on the levels of diversity supported by plantations. We suggest that a wide range of scales should be considered in the evaluation of the role of forest plantations on biodiversity conservation.

## Introduction

Forests are vital to the conservation of terrestrial biodiversity, as more than a quarter of the earth's land surface is covered by forests, and more than half of the earth's terrestrial species dwell in or depend on these forests [1], [2]. However, in many regions, the ratio of natural forest is low due to anthropogenic activities [3], which has made modified forests an important component in preventing the loss of biodiversity [4]. Planted forests, a subset of modified forests, are being expanded in area annually to combat the global trend in shrinking forest cover [2]. The role of forest plantations in supporting biodiversity is critically important in regions where natural forests have become highly modified [5], [6]. However, knowledge about plantations in biodiversity conservation is incomplete and their contribution to such conservation is still arguable [7], [8], [9], [10], [11].

The value of forest plantations in biodiversity conservation has been found to be context dependent [7], [12], [13], [14]. In their recent work, Bremer and Farley [9] used 126 observations from 36 published works to conduct a quantitative synthesis of the effects of forest plantations on plant richness. They found that the effects of plantations on plant richness varied considerably depending on whether the original land cover was grassland, shrub land, primary forest, secondary forest, or degraded or exotic pasture, and whether native or exotic tree species had been planted. They suggested that plantations have positive effects on plant diversity when established on degraded lands by comparison to when they are used to replace natural ecosystems (such as forests, grasslands, and shrub lands), and when indigenous rather than exotic tree species are used [9]. These results provide useful information with respect to which land types and species should be selected for afforestation in order to achieve optimal biodiversity outcomes. However, the cases selected for their analysis focused on only one spatial scale [9], as has almost all previous research. In fact, Brockerhoff et al. [7] and Hartmann et al. [15] pointed out that the negative or positive effects of plantations on biodiversity are scale dependent. However, to the best of our knowledge, there have been no attempts to address this scale dependency issue. There is an urgent need for multi-scale evaluations to assess the influence of planted forests on biodiversity and to identify the appropriate spatial scale at which management efforts should be implemented in order to obtain favorable biodiversity outcomes.

The rationale for such assessments is that biodiversity distribution in heterogeneous landscapes is the result of different processes operating at different scales [16], [17], such that the effects of these processes on the distribution pattern of biodiversity may change with scale. A multi-scale analysis of diversity makes it possible to identify the spatial scale at which a forest plantation has a positive (or negative) effect on biodiversity and, therefore, the appropriate spatial scale at which the management strategy for an optimal biodiversity outcome should be designed [18], [19], [20]. The additive diversity partitioning approach is an ideal candidate for the quantification of diversity across multiple spatial scales [21], [22], [23], [24], [25].

In the additive diversity partitioning approach, the total species diversity (gamma diversity, *γ*) is the sum of within-community diversity (alpha diversity, α) and between-community diversity (beta diversity, *β*), i.e., *γ* = *α*+*β* [21], [26], [27]. This approach treats alpha diversity as the average within-community diversity, regardless of whether diversity is measured by species richness or Simpson's diversity index. Beta diversity is thus not the average amount of diversity found in a single, randomly chosen unit, or the average diversity missing from a community, but that which is present in the total diversity of all other communities in the assemblage. Therefore, alpha and beta diversity are commensurate and can be compared directly, so that the partitioning can be applied at any spatial scale [24].

In our study, to partition patterns of woody species diversity, we designed a hierarchical sampling system including four levels: plot, slope position, slope, and watershed (corresponding to different sampling scales). The watershed is a basic geohydrological unit in a mountainous region that is used for effective ecological management practices to sustain and enhance ecosystem functions [28]. Slope and slope position were selected as a level of analysis in the hierarchical sampling system because these factors can determine the distribution patterns of species [29]. Furthermore, these factors could potentially be used in forest management strategies to select the location of plantations. To analyze the effects of forest plantations on woody species diversity, we selected 9 small watersheds, 3 were planted pine (*Pinus tabuliformis* Carr.) forest, 3 were planted larch (*Larix principis-rupprechtii* Mayr.) forest, and 3 were natural secondary broadleaf forest. We hypothesized that the effects of forest plantations on woody species diversity (tree, shrub, and regeneration layers) would vary with sampling scales. We compared the multi-scale partitioning patterns of woody species diversity using the additive diversity partitioning approach. We tested and compared the difference of all species diversity components (alpha, beta, and gamma diversity) among forest types using mixed-effect models and multiple comparisons.

## Methods

### Ethics statement

No specific permits were required for the described field studies. The location was not privately-owned or protected in any way, and the study did not involve endangered or protected species.

### Study area

The study was conducted on Donglingshan Mountain, an extension of the Xiaowutaishan Mountains belonging to the broader Taihangshan Mountains, 100 km northwest of Beijing City, China. The study area, within Beijing Xiaolongmen National Forest Park, is located at 39°57'53" N and 115°26'05" E. The soil type in the area is brown soil, classified as Eutriccambisol [30]. The area has a typical warm, temperate, continental monsoon climate with an average annual precipitation of 500–650 mm. The mean annual temperature is 5–10°C. The altitude of most of the area is greater than 1000 m above sea level, with the highest peak at 2303 m.

The zonal vegetation is highly heterogeneous, warm temperate zone deciduous broadleaf forest [31], including primarily oaks (*Quercus* spp.), mixed species (e.g., *Tilia* spp., *Ulmus* spp., *Acer* spp., *Juglans mandshurica* and *Fraxinus rhynchophylla*), birches (*Betula* spp.) and poplar (*Populus davidiana*). There are also pine (*P. tabuliformis* Carr.) and larch (*L. principis-rupprechtii* Mayr.) forest plantations that were established during the late1950s. During this period, most natural broadleaf forests were clear-cut for the steel industry. After deforestation, some of the small watersheds in the area were planted with pines (*P. tabuliformis* Carr.), while others were planted with larch (*L. principis-rupprechtii* Mayr.); still others are secondary broadleaf forest that recovered naturally. The primary purpose of the plantations has been for wood production. Now, since the establishment of the nature reserve in 1985, which then became a forest park in 1994, all of the forests in this area—both natural and planted—serve roles in biodiversity conservation, soil and water conservation, and carbon sequestration [32].

### Sampling design and data collection

**Figure 1:**
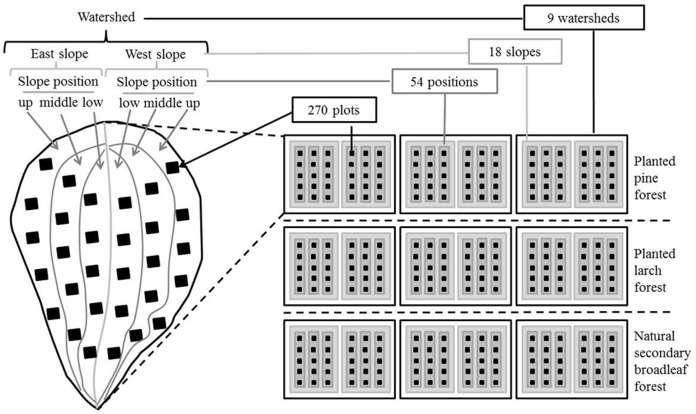
Hierarchical nested sampling design used for data collection. The hierarchical sampling system included 4 levels (corresponding to spatial scales): 270 sampling units at the plot scale were nested within 54 sampling units at the slope position scale; 54 sampling units at the slope position scale were nested within 18 sampling units of slope scale, and 18 sampling units at the slope scale were nested within 9 sampling units at the watershed scale. For the 9 watersheds, 3 watersheds were planted in pine forest, 3 in larch forest, and 3 were natural secondary broad-leaved forest.

We designed a hierarchically nested sampling system to collect data on woody species diversity in the study area. The system included 4 hierarchical levels: plot, slope position, slope, and watershed (corresponding to spatial scales). The highest level was the watershed. In this region, most watersheds have only two main slope exposures, with areas ranging in size from 20–30 ha. We selected 9 such watersheds, with primarily eastern and western slope exposures: 3 were pine (*P. tabuliformis* Carr.) plantations, 3 were larch (*L. principis-rupprechtii* Mayr.) plantations, and 3 were natural secondary broadleaf forests (recovered naturally after the clear-cutting). The natural secondary broadleaf forest selected in this work was ideal as the control for the comparison of the effects of plantations on woody species diversity. In each watershed, two slopes (eastern and western exposures) were nested; in each slope, three slope positions (upper, middle, lower) were nested; and in each slope position, 5 10 m×10 m plots were selected for data collection (Fig. 1). In each plot, species were recorded separately at the tree, shrub, and regeneration layers. In the tree layer, the species name, height, and coverage of all individual trees with a height of more than 3 m were recorded; in the shrub layer, the species name, abundance, height, and coverage of all shrub species were recorded, and in the regeneration layer, the species name, height, and coverage of all individual trees smaller than 3 m were recorded. In total, 30 plots (5 plots/slope positions ×3 slope positions/slope ×2 slopes/watershed) were collected in each watershed. A total of 270 plots were collected, comprising 54 samples of slope position, 18 samples of slope, and 9 samples of watershed for the data analysis (Fig. 1). All field work was conducted during the summer (July and August) of 2011 and 2013.

### Multi-scale partitioning of diversity

**Figure 2:**
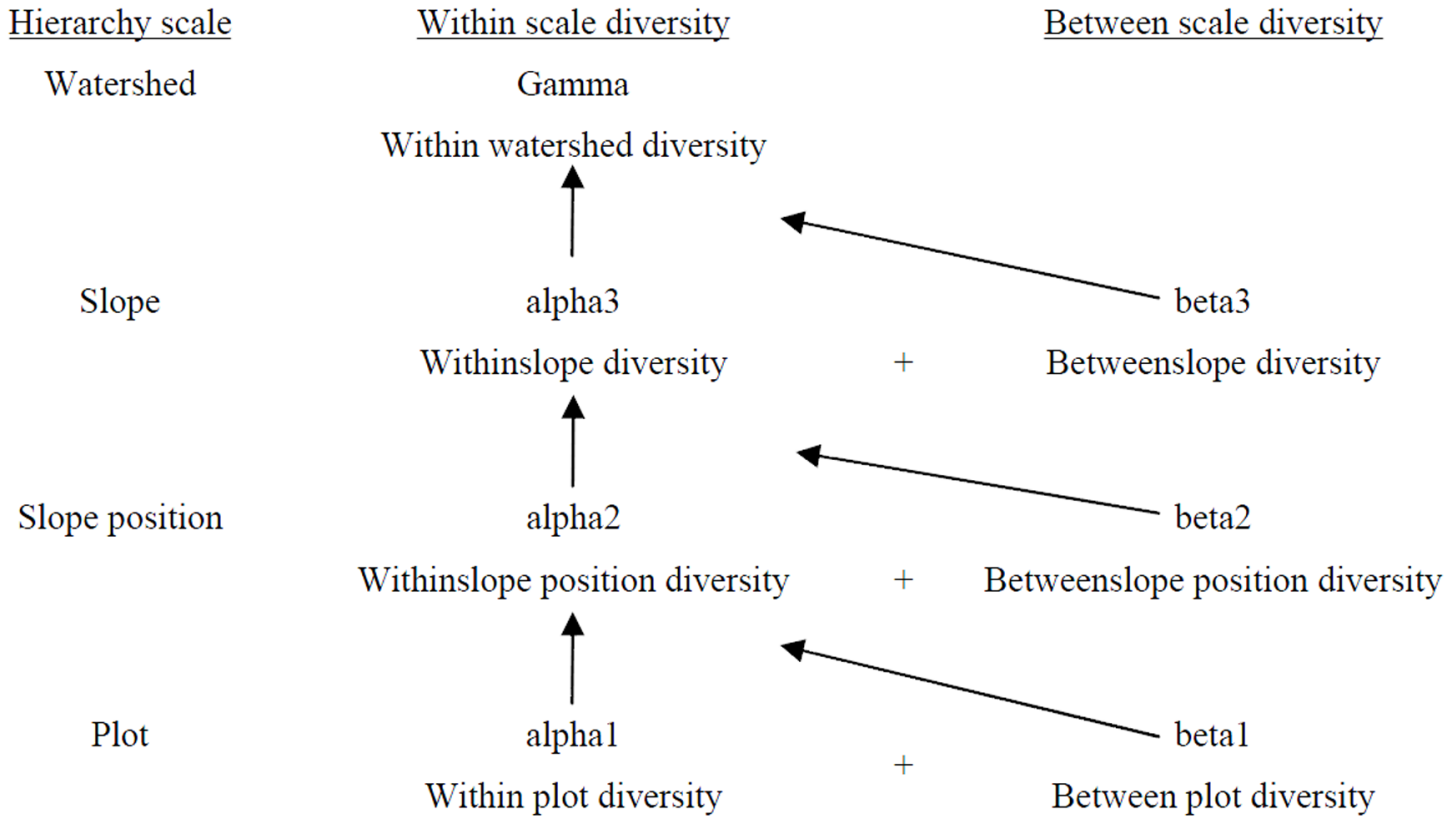
Hierarchical levels in the additive partitioning of diversity used in the study: the diversity of each scale was linked additively to form the diversity of the next higher scale (adapted from Wagner et al., 2000; Gering et al., 2003; Chávez and Macdonald, 2012).

It is well known that there are two different perspectives in diversity partitioning: multiplicative and additive [33]. There has been extensive discussion of which perspective is better for partitioning “independent” beta diversity (see a recent Forum in *Ecology* edited by Ellisonand papers in it [33]), but both perspectives have advantages and disadvantages [34], [35]. In the additive partitioning approach, all diversity components have the same unit, which makes it possible to compare different diversity components at multiple scales [21]. Therefore, we used the additive approach in our work. According to the hierarchical model of additive partitioning, gamma diversity (the diversity in a watershed) can be partitioned into its alpha and beta components at four spatial scales (Fig. 2). In each hierarchical level, we partitioned the total woody species diversity observed in the tree, shrub, and regeneration layers into within-scale (alpha diversity) and between-scale diversity (beta diversity). At the lowest sampling level, the plot level, the within-plot diversity (alpha1) is the mean species diversity in a plot (per plot in a slope position level) and the between-plot diversity (beta1) is determined by subtracting the within-plot diversity (alpha1) from the species diversity in pooled plots (all 5 plots per slope position). Similarly, the within-slope position diversity (alpha2) is the mean species diversity in the slope position (per slope position on a slope) and the between-slope position diversity (beta2) is determined by subtracting the within-slope position diversity (alpha2) from the species diversity in the pooled slope position (all 3 slope positions per slope). The within-slope position diversity (alpha3) is the mean species diversity in a slope (per slope in a watershed) and the between-slope diversity (beta3) is determined by subtracting the within-slope position diversity (alpha3) from the species diversity in pooled slopes (2 slopes per watershed, resulting in the gamma diversity). Thus, the total diversity (gamma diversity) in the watershed can be partitioned as: gamma  =  alpha1+ beta1+ beta2+ beta3.

Species richness and Simpson's diversity index were selected as the two measures of diversity for woody species in this work. Previous studies [36], [37] have suggested that species richness recovers much faster than species composition in secondary forests or plantation forests. Species richness reflects only the presence or absence of a species in a community (or ecological assemblage), while Simpson's diversity index can reflect the species composition of a community (or ecological assemblage). The Simpson's diversity index is calculated by the following equation [38]:



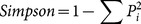
(1)where *P_i_* (calculated by [*h_i_*/*th*+*c_i_*/*tc*+*a_i_*/*ta*]/3) is the important value of species *i*, which is used primarily for the calculation of the diversity measure in plant communities [39]; *h_i_*, *c_i_*, and *a_i_* are the sum of the height, coverage and number of individuals for species *i*; *th*, *tc*, and *ta* is the total value of the height, coverage, and number of all individuals for all species, respectively.

### Statistical analysis

The variability in different diversity components (alpha1, alpha2, alpha3, beta1, beta2, beta3, gamma; see S1 Appendix for the data used in this study) for each layer (tree, shrub, and regeneration layer) was analyzed using the mixed-effect model, which accounts for non-independent errors caused by the hierarchically nested sampling method [20], [36], [40]. According to the diversity components used for analysis, forest type, slope, and slope position, as well as their first-order interactions, were set as fixed effects; the watersheds, which differ in area and environmental conditions that may cause differences in species diversity were set as random effects, while slope nested in watershed, and slope position nested in slope, were set initially as random effects (Table 1). The best-fit models for all diversity components in the different woody species layers were determined by likelihood ratio tests in two steps: first, the random effect variables simplification was performed. The random effects were dropped from the highest hierarchical sampling level (watershed) to the lowest sampling level step by step until the drop produced a significant decrease in the model's likelihood [37]; second, the fixed effects variables simplification was conducted. After the simplification of random effects variables, the non-significant fixed effects variables or interactions were dropped from the model step by step until, when dropped, all variables included in the model produced a significant decrease in the model's likelihood. However, the non-significant variables included in significant interactions were not removed from the models [41], [42]. The AIC was used in the selection of the best fitted model. The significance of fixed effect was calculated using ‘lmerTest’ package[43] and the linear mixed model was fitted by ‘lme4’package [44] in R. Multiple comparisons for the fixed effects were conducted with the statistical package “multcomp” which can calculate simultaneous tests and confidence intervals for general linear hypotheses in mixed- effect models in R. The Tukey test was used for all pairwise comparisons [45]. We conducted multiple comparisons for forest type, first-order interactions for forest type and slope, first-order interactions for forest type, and slope and slope position in diversity components alpa1 and beta1only. All data were square-root transformed (richness) or natural-log transformed (Simpson's diversity index) prior to statistical analysis [46]. All analyses were conducted using software R [47].

**Table 1:** Model information of the mixed-effects models used for different diversity components.

Diversity components	Fixed effect	Random effect
alpha1, beta1	Forest type, slope, slope position, forest type × slope, forest type × slope position, slope × slope position, forest type × slope × slope position	Watershed/slope/slope position
alpha2, beta2	Forest type, slope, forest type × slope	Watershed/slope
alpha3, beta3, gamma	Forest type	Watershed

## Results

**Figure 3:**
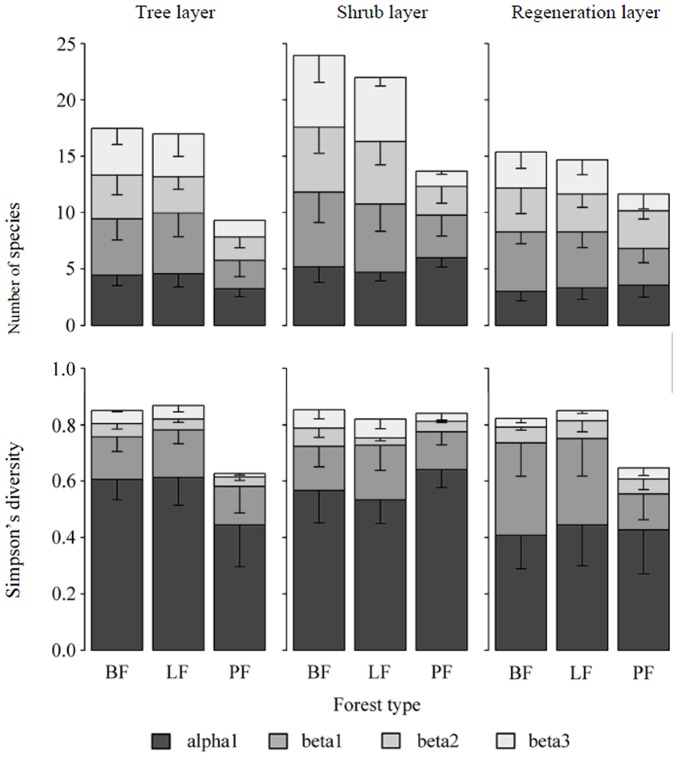
Components of woody species diversity (mean alpha diversity at the plot scale, beta diversity at the plot, slope position, and slope exposure scales, and gamma diversity at the watershed scale) in the tree, shrub, and regeneration layers for different forest types. PF-planted pine forest; LF-planted larch forest; BF-natural secondary broadleaf forest.

A total of 70 woody species were recorded at the study site, of which 31 were in the tree layer, 39 were in the shrub layer and 27 were in the regeneration layer. There was a total of 58 species recorded in the natural secondary broadleaf forest, 27 in the tree layer, 31 in the shrub layer, and 20 in the regeneration layer. In the larch forest plantation, the total number of species recorded was 57, 22 in the tree layer, 35 in shrub layer, and 19 in regeneration layer. In the pine forest plantations, the corresponding numbers were 32, 15, 17, and 17. With respect to the multi-scale partitioning pattern (Fig. 3), in the pine plantations, the contribution sequence to a watershed's species richness was alpha1> beta1> beta2> beta3 for all three layers, while the sequence in natural secondary broadleaf forests and larch plantations was beta1> beta3> beta2> alpha1 in the tree and shrub layers and beta1> beta2> beta3> alpha1 in the regeneration layer. For the Simpson's diversity, the highest contribution to the watershed diversity was alpha1 in all three layers for all forest types; the contribution sequence of the beta diversity components for pine forest plantations was beta1> beta2> beta3 in all three layers; the sequence in natural secondary forests and planted larch forests was the same as in planted pine forests in the regeneration layer, but in the tree and shrub layers, the sequence was beta1> beta3> beta2.

The results of the mixed-effects models for within-scale diversity components (alpha1, alpha2, alpha3, and gamma) are shown in Table 2. In the tree layer, the forest types showed significant differences in species richness and Simpson's diversity for all within-scale diversity components. In the shrub layer, the forest types showed significant differences in species richness at the plot and watershed sampling scales (alpah1and gamma), while they showed significant differences in Simpson's diversity only at the plot sampling scale (alpha1). In the regeneration layer, the forest types showed significant differences in species richness and Simpson's diversity only at the slope position sampling scale (alpha2). Slope had significant effects on species richness in the tree layer and on Simpson's diversity in the regeneration layer at the plot sampling scale (alpha1). The first-order interactions of forest type and slope showed significant differences in species richness at the plot scale (alpha1) in the tree layer, while the first-order interactions of forest type and slope position showed significant differences in species richness at the plot sampling scale (alpha1) in the tree layer.

**Table 2:** Best-fit linear mixed-effect models testing the effects of forest type, slope, slope position, and first-order interactions on alpha diversity and gamma diversity components.

Diversity components	Source of variation	*df*	Richness			Simpson's diversity		
			Tree	Shrub	Regen	Tree	Shrub	Regen
**Alpha1**	**Forest type**	2	13.0**	4.0*	0.6	17.4***	4.1*	0.9
	**Slope**	1	5.0*			3.8		4.4*
	**Slope position**	2	3		0.7			1.1
	**Forest type × Slope**	2	6.0*			3.1		
	**Forest type × Slope position**	4			4.1**			2.4
	**Slope× Slope position**	2						
	**Forest type × Slope× Slope position**	4						
**Alpha2**	**Forest type**	2	6.9**		5.0*	16.8***		6.2*
	**Slope**	1			0.0	3.4		2.9
	**Forest type × Slope**	2			3.0	2.8		4.1
**Alpha3**	**Forest type**	2	8.3*			25.4**		
**Gamma**	**Forest type**	2	33.0***	7.5*		38.3***		

Notes: F values of parameters included in best-fit models for each response variable are given. Empty cells indicate parameters that were considered initially in models but finally were not included in best-fit models according to log-likelihood tests. Tree-tree layer; Shrub-shrub layer; regen-regeneration layer; * *p*<0.05; ** *p*<0.01; *** *p*<0.001.

**Figure 4:**
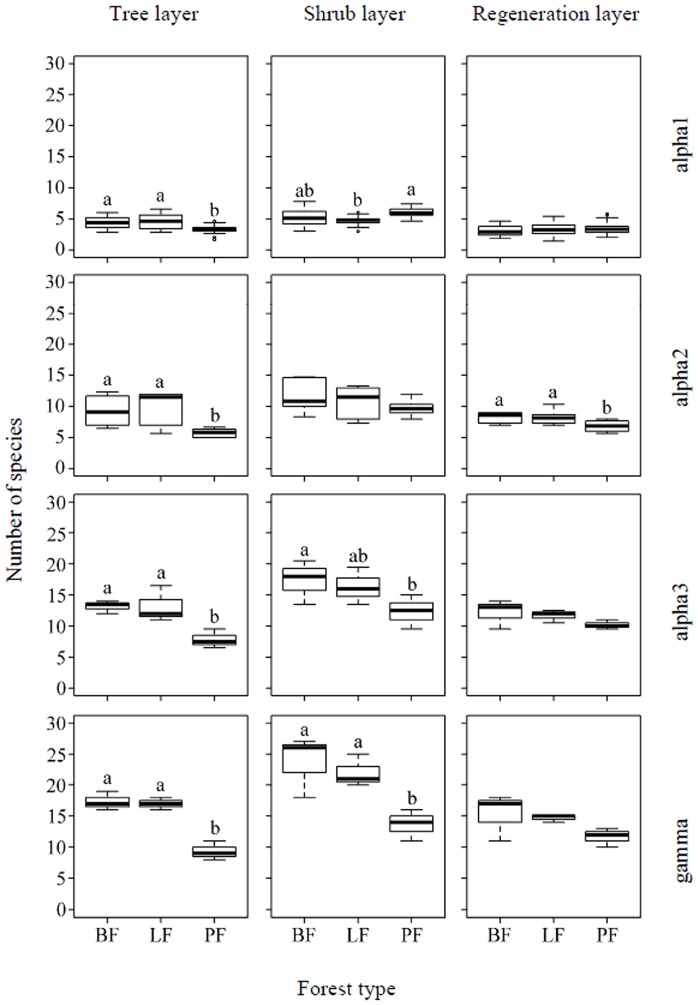
Box plots of alpha diversity (alpha1, alpha2, and alpha3) and gamma diversity components measured by species richness of the tree, shrub, and regeneration layers in different forest types. The center lines represent medians, and the outer lines represent the inter-quartile range. Whisker lines represent the whole range of data that lie within one and a half times the inter-quartile range (1.5×IQR). PF, LF, BF: see Fig. 3.

The results of the mixed-effects models for between-scale diversity components (beta1, beta2, beta3; Table 3) showed significant differences in beta diversity measured by species richness at the plot sampling scale (beta1) and significant differences in beta diversity measured by Simpson's diversity were found at the slope sampling scale (beta3) for forest type in the tree layer. In the shrub layer, beta diversity measured by species richness was significant for forest type at the slope sampling scale (beta3). For beta diversity measured by Simpson's diversity, significant differences among forest types were found at the slope position sampling scale (beta2). In the regeneration layer, significant differences in the beta diversity (measured both by species richness and Simpson's diversity) were found at the plot sampling scale (beta1) for all forest types. Slope position demonstrated significant differences at the plot sampling scale (beta1) in the tree and shrub layers for beta diversity measured by species richness. First-order interactions for forest type and slope position showed significant differences at the plot sampling scale (beta1) in the regeneration layer for beta diversity measured by species richness. The differences in the within-scale diversity components among forest types are shown in Figs. 4 (species richness) and 5 (Simpson's diversity). Compared to the other forest types, species diversity (species richness and Simpson's diversity) in the tree layer in the pine plantations was significantly lower than in the natural secondary broadleaf forests and larch plantations for all within-scale diversity components. In the shrub layer, the species richness in the pine forest plantations was significantly lower than in the natural secondary broadleaf forests and planted larch forests at slope (alpha3) and watershed (gamma) sampling levels, but the species richness was significantly higher than in the planted larch forests at the plot (alpha1) sampling level; the Simpson's diversity in the planted pine forests was significantly higher than in the planted larch forest at the plot (alpha1) and slope (alpha3) sampling levels. In the regeneration layer, the species richness in the pine forest plantations was significantly lower than in the natural secondary broadleaf forests and larch forest plantations at the slope position (alpha2) sampling scale; the Simpson's diversity in planted pine forests was significantly lower than in the natural secondary broadleaf forests and planted larch forests at the slope position (alpha2), slope (alpha3) and watershed (gamma) sampling scales.

**Table 3:** Best-fit linear mixed-effect models testing the effects of forest type, slope, slope position, and first-order interactions on beta diversity components.

Diversity components	Source of variation	*df*	Richness			Simpson's diversity		
			Tree	Shrub	Regen	Tree	Shrub	Regen
**Beta1**	**Forest type**	2	8.2**	3.6	17.4***			9.4*
	**Slope**	1			1.0			
	**Slope position**	2	8.0**	7.4**	2.6			
	**Forest type × Slope**	2			3.9			
	**Forest type × Slope position**	4			2.8*			
	**Slope× Slope position**	2						
	**Forest type × Slope× Slope position**	4						
**Beta2**	**Forest type**	2					4.5*	5.4*
	**Slope**	1						
	**Forest type × Slope**	2						
**Beta3**	**Forest type**	2		19.8**		9.2*		

Notes: F values of parameters included in best-fit models for each response variable are given. Empty cells indicate parameters that were considered initially in models but finally were not included in best-fit models according to log-likelihood tests. Tree-tree layer; Shrub-shrub layer; regen-regeneration layer; * *p*<0.05; ** *p*<0.01; *** *p*<0.001.

The differences in the between-scale diversity components among forest types are shown in Figs. 6 (species richness) and 7 (Simpson's diversity). In the tree layer, the species richness in the pine forest plantations was significantly lower than in the natural secondary broadleaf forests and larch forest plantations at the plot (beta1) and slope (beta3) sampling levels, but was only significantly lower than in the natural secondary broadleaf forests at the slope position (beta2) sampling scale; the Simpson diversity in the planted pine forests was significantly lower than in the planted larch forests at the plot (beta1) sampling level, and was significantly lower than in the natural secondary broadleaf forests and planted larch forests at the slope (beta3) sampling scale. In the shrub layer, the species richness in the pine forest plantations was significantly lower than in the natural secondary broadleaf forests and larch forest plantations at the slope position (beta2) and slope (beta3) sampling levels, but was significantly lower than in the planted larch forests only at the slope position (beta1) sampling scale; the Simpson diversity in the planted larch forests was significantly lower than in the natural secondary broadleaf forests at the slope position (beta2) sampling level. In the regeneration layer, species diversity (both species richness and Simpson's diversity) in the planted pine forests was significantly lower than in the natural secondary broadleaf forests and planted larch forests only at the plot (beta1) sampling level.

The multiple comparisons of first-order interactions for forest type and slope at the plot sampling scale (alpha1 and beta1) revealed 13 comparable pairs that were significantly different. For alpha diversity (alpha1), the species richness on the western exposure of a slope in the pine plantations was significantly lower than in the natural secondary broadleaf forest (estimated effects: −0.39±0.09 [mean ± SE], z = −4.16, *p*<0.001) for the tree layer; the species richness on the eastern exposure of a slope in the planted pine forests was significantly lower than in the planted larch forest (estimated effects: −0.51±0.09, z = −5.44, *p*<0.001), while the species richness on the eastern exposure of a slope in the planted larch forest was significantly higher than in the natural secondary broadleaf forest (0.27±0.09, z = 2.91, *p* = 0.042) for the tree layer; the Simpson diversity on the western exposure of a slope in the planted pine forest was significantly lower than in the natural secondary broadleaf forest (estimated effects: −0.43±0.08, z = −5.31, *p*<0.001) and the planted larch forest (estimated effects: −0.34±0.08, z = −4.20, *p*<0.001) for the tree layer; the Simpson diversity on the eastern exposure of a slope in the planted pine forest was significantly lower than in the planted larch forest (estimated effects: −0.31±0.08, z = −3.81, *p*<0.01) for the shrub layer. For beta diversity, the species richness on the eastern exposure of a slope in the pine forest plantations was significantly lower than in the larch forest plantations for all three layers (estimated effects: −0.96±0.24, z = −4.05, *p*<0.001 for the tree layer; −0.88±0.25, z = −3.51, *p* = 0.006 for the shrub layer; −0.67±0.13, z = −5.05, *p*<0.001 for the regeneration layer); the species richness in the pine forest plantations was significantly lower than in the natural secondary broadleaf forest on both slope exposures (estimated effects: −0.58±0.13, z = −4.37, *p*<0.001 for the eastern exposure; −0.42±0.13, z = −3.04, *p* = 0.029 for the western exposure) in the regeneration layer; the Simpson diversity on the eastern exposure of a slope in the pine forest plantations was significantly lower than in the larch forest plantations (estimated effects: −0.60±0.20, z = −3.04, *p* = 0.028 for the shrub layer); the Simpson's diversity on the western exposure of a slope in the planted pine forest was significantly lower than in the natural secondary broadleaf forest (estimated effects: −1.76±0.38, z = −4.67, *p*<0.001) and planted larch forest (estimated effects: −1.52±0.37, z = −4.12, *p*<0.001).

The multiple comparisons of first-order interactions for forest type, slope, and slope position at the plot sampling scale (alpha1 and beta1) showed 11 comparable pairs that were significantly different. For alpha diversity, species richness at the middle slope position on the eastern exposure of a slope in the planted pine forest was significantly lower than in the planted larch forest (estimated effects: −0.51±0.13, z = −3.83, *p* = 0.014); the species richness at the upper slope position on the western exposure of a slope in the planted pine forest was significantly lower than in the natural secondary broadleaf forest (estimated effects: −0.71±0.14, z = −5.17, *p*<0.001); the Simpson diversity at the upper slope position on the western exposure of a slope in the planted pine forest was significantly lower than in the planted larch forest (estimated effects: −0.70±0.12, z = −5.79, *p*<0.001) and natural secondary broadleaf forest (estimated effects: −0.53±0.12, z = −4.35, *p*<0.01) in the tree layer; in the shrub layer, however, the Simpson diversity at the lower slope position on the eastern exposure of a slope in the planted pine forest was significantly higher than in the natural secondary broadleaf forest (estimated effects: 0.44±0.12, z = 3.57, *p* = 0.037). For beta diversity, in the regeneration layer, the species richness at the middle slope position on the eastern exposure of a slope in the pine forest plantations was significantly lower than in the larch forest plantations (estimated effects: −0.83±0.19, z = −4.48, *p*<0.01) and natural secondary broadleaf forest (estimated effects: −0.80±0.19, z = −4.32, *p*<0.01); the species richness at the middle slope position on the western exposure of a slope in the pine forest plantations was significantly lower than in the natural secondary broadleaf forest (estimated effects: −0.83±0.19, z = −4.48, *p*<0.001); the species richness at the upper slope position on the eastern exposure of a slope in the pine forest plantations was significantly lower than in the larch forest plantations (estimated effects: −0.72±0.19, z = −3.88, *p* = 0.013); the Simpson diversity at the middle slope position on the western exposure of a slope was significantly lower than in the larch forest plantations (estimated effects: −2.17±0.49, z = −4.38, *p*<0.01) and natural secondary broadleaf forest (estimated effects: −2.30±0.49, z = −4.66, *p*<0.001).

## Discussion

The additive diversity partitioning method is a powerful approach in diversity pattern analysis across spatial scales [19], [20], [23], [42], [46]. We assessed the effects of forest plantations on patterns of woody species diversity partitioning with this hierarchical multi-scale approach. Our work showed that the multi-scale diversity partitioning patterns in a watershed differed among forest types. In a watershed, the pine plantations (*P. tabuliformis* Carr.) changed the multi-scale diversity partitioning pattern of woody species diversity (except the Simpson diversity in the regeneration layer) compared to natural secondary broadleaf forest, while the larch plantations (*L. principis-rupprechti*) did not show multi-scale diversity partitioning patterns that were obviously different from those in the natural secondary broadleaf forest (Fig. 3). Our results highlight the fact that forest plantations affect plant diversity at multiple scales, thereby reemphasizing the necessity of taking multiple spatial scales into account in the assessment of the effects of forest plantations on biodiversity conservation.

**Figure 5:**
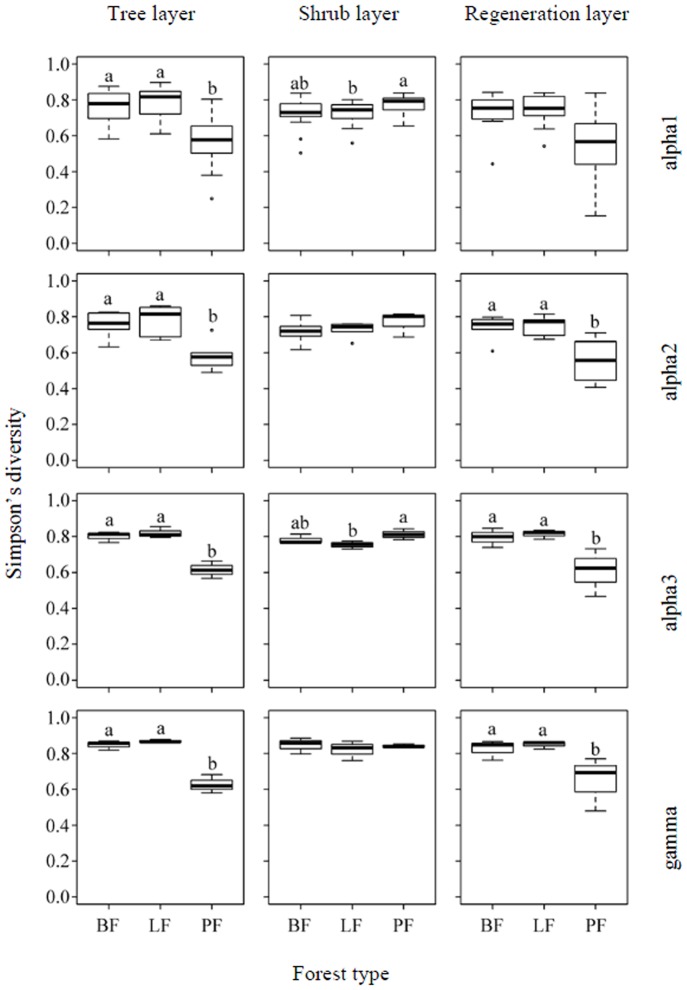
Box plots of alpha diversity (alpha1, alpha2, and alpha3) and gamma diversity components measured by Simpson's diversity of the tree, shrub, and regeneration layers in different forest types. The center lines represent medians, and the outer lines represent the inter-quartile range. Whisker lines represent the whole range of data that lie within one-and-a-half times the inter-quartile range (1.5×IQR). PF, LF, BF: see Fig. 3.

**Figure 6:**
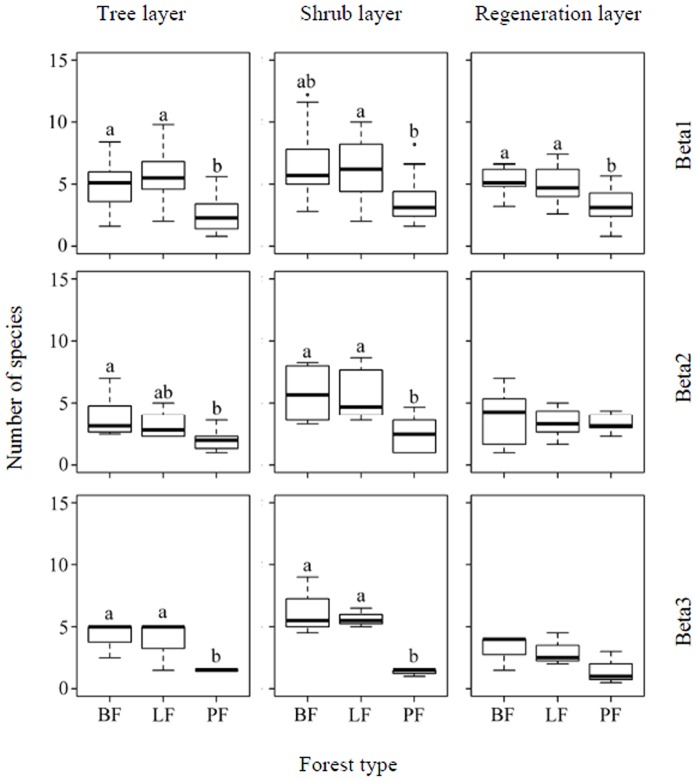
Box plots of beta diversity components (beta1, beta2, and beta3) measured by species richness of the tree, shrub, and regeneration layers in different forest types. The center lines represent medians, and the outer lines represent the inter-quartile range. Whisker lines represent the whole range of data that lie within one and a half times the inter-quartile range (1.5×IQR). PF, LF, BF: see Fig. 3.

**Figure 7:**
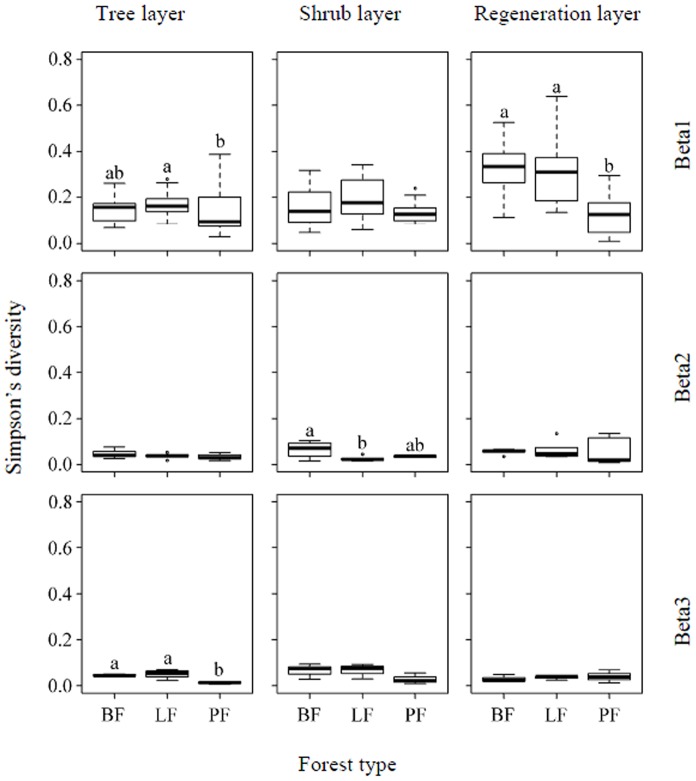
Box plots of beta diversity components (beta1, beta2, and beta3) measured by Simpson's diversity of the tree, shrub, and regeneration layers in different forest types. The center lines represent medians, and the outer lines represent the inter-quartile range. Whisker lines represent the whole range of data that lie within one-and-a-half times the inter-quartile range (1.5×IQR). PF, LF, BF: see Fig. 3.

Our work verified, in part, our hypothesis that the effects of forest plantations on woody species diversity depend on the sampling scale selected. For within-diversity components (alpha1, alpha2, alpha3, gamma), the significance of the difference among forest types was dependent on the sampling scale selected in the shrub and regeneration layers, although not in the tree layer which showed significant differences among forest types for all within-diversity components (Table 2, Figs. 4 and 5). For between-diversity components (beta1, beta2, beta3), the significance of the difference was dependent for all layers (Table 3, Figs. 6 and 7). This is the first work that provides direct evidence for the recent hypothesis that the effects of forest plantations on diversity might be scale-dependent [7], [15]. When structuring plant communities, both biotic and abiotic factors have been shown to affect different processes at varying scales [16], [17]; this implies that their effects on species diversity may change with scale.

Previous work has shown that the effects of forest plantations are also dependent on the species selected [9]. Our results were consistent with those studies, in that the larch plantations (*L. principis-rupprechtii*) did not have significant difference from the natural secondary broadleaf forest, while the pine plantations (*P. tabuliformis*) had different effects on woody species richness at different sampling scales compare to natural secondary broadleaf forest (Figs. 4–7). This suggests that the characteristics of a dominant species play a critical role in plantations with respect to species diversity. Compared to larch (*L. principis-rupprechtii*) and broadleaf deciduous species, pine (*P. tabuliformis*) is an evergreen, which changes seasonal light schemes, especially during the non-growing season, ultimately increasing the homogeneity of the light environment for woody species. As biotic homogenization has been suggested to be a threat to biodiversity [48], it is not surprising that the value of beta diversity components in the planted pine forest were nearly the lowest among the forest types (except beta2 in the shrub layer measured by Simpson's diversity; Figs. 6 and 7).

In most regions, forest plantations were designed for degraded ecosystem recovery and biodiversity conservation [7], [15]. The results of our work showed that the pine forest plantations in watersheds change the diversity pattern of woody species (but not for the Simpson diversity in the regeneration layer) and decrease woody species diversity (the significant effects showed at different sampling scales), while a watershed planted with larch does not change the diversity pattern or decrease the diversity compared to the natural forests. In a watershed, slope exposure and slope position are very important indirect factors governing the output of diversity [29]. When we take the slope exposure into consideration, the larch plantations had a significantly higher value of woody species diversity (alpha diversity in the tree layer, beta diversity measure by species richness for all layers, beta diversity measured by Simpson's diversity at the shrub layer) than did the pine plantations on the eastern exposure of a watershed. On the western exposure slope of a watershed, our work showed that the pine plantations lowered the alpha diversity in the tree layer and beta diversity in the regeneration layer significantly by comparison to the natural secondary broadleaf forest. When slope exposure and position were both taken into consideration, we found that the pine plantations decreased the alpha diversity at the upper slope position in the tree layer and the beta diversity at the middle slope position in the regeneration layer on the western exposure of the slope significantly, while on the eastern exposure of a slope, the pine plantations at the middle slope position significantly decreased alpha diversity in the tree layer compare to the larch plantations and beta diversity in the regeneration layer compare to the larch plantations natural secondary broadleaf forests, but increased the alpha diversity (Simpson's diversity) at the lower slope position significantly compared to the natural secondary broadleaf forests. These results suggest that slope exposure and position are important in assessing the woody species diversity of plantations in mountainous regions. These results have potential practical value in guiding afforestation plans for this region. When the minimum management unit for the afforestation plan is the watershed, our work suggests that larch plantations or natural recovery of broadleaf forest are preferable. When the minimum management unit scale is reduced to the slope level, larch plantations are recommended on the eastern exposure rather than pine plantations. When the minimum management unit scale is reduced further to the slope position level, pine plantations in the upper and middle slope positions on western exposures, and in the middle slope position on eastern exposure of a slope are not recommended; instead, the natural forest should be allowed to recover.

There is increasing recognition that ecosystems and their services need to be managed in the face of landscape change [49]. Forest plantations, a unique land type used to potentially counteract the worldwide decline in forested area [2], play critical roles in maintaining ecological functions, especially in mountainous regions. The watershed is not only a basic geohydrological unit, but also a unit for forest management in mountainous areas [28]. With this new multi-scale strategy, focus has to be pointed at the impact of biodiversity within the watershed of planted forest. Our study points to the importance of sampling scale and species selection in assessing the influence of plantations on woody species diversity in afforestation area.

## Supporting Information

S1 Appendix **Data used in this study**.
(XLSX)
